# 966. Large-Scale Evaluation of AST Prediction using Resistance Marker Presence/Absence vs. Machine Learning on WGS Data

**DOI:** 10.1093/ofid/ofad500.2461

**Published:** 2023-11-27

**Authors:** Arolyn Conwill, Mohamad Sater, Nicholas Worley, Jason Wittenbach, Miriam H Huntley

**Affiliations:** Day Zero Diagnostics, Boston, Massachusetts; Day Zero Diagnostics, Boston, Massachusetts; Day Zero Diagnostics, Boston, Massachusetts; Day Zero Diagnostics, Boston, Massachusetts; Day Zero Diagnostics, Boston, Massachusetts

## Abstract

**Background:**

Whole genome sequencing (WGS) of bacterial pathogens directly from clinical samples or cultured isolates is a promising technique for diagnosing infections, yet challenges remain in using WGS to guide antimicrobial therapy. The standard approach predicts antimicrobial susceptibility test (AST) profiles using the presence/absence of known resistance markers. Here we measure the performance of resistance markers compared to a machine learning method for predicting AST.

**Methods:**

We assessed ResFinder, a publicly available bioinformatics tool that detects a curated set of resistance genes and point mutations, to test the utility of resistance marker presence or absence for predicting resistance/susceptibility on 107 species/drug combinations relevant for bloodstream infections. We also tested the performance of Keynome *g*AST, a machine learning method we developed that predicts AST from WGS data using the entirety of the bacterial genome without limiting to known resistance markers. Performance was assessed on >36,000 bacterial strains from MicrohmDB®, a large-scale database containing WGS data and phenotypic AST results for tens of thousands of clinical bacterial isolates.

**Results:**

ResFinder performance varied widely across species/drug combinations, with a median balanced accuracy of 80% (52%-92%, 1st and 3rd quartiles). ResFinder detected a resistance marker in only 72.3% of resistant and 50.3% of intermediate sample-drug combinations, indicating that many phenotypically nonsusceptible organisms may be mischaracterized by relying on resistance marker presence. Conversely resistance markers were present in 14.2% of susceptible sample-drug combinations. In contrast, across the same species/drug combinations Keynome *g*AST had a median balanced accuracy of 92% (87%-96%, 1st and 3rd quartiles), and was superior to ResFinder in 69% and equivalent to ResFinder in 28% of species/drug combinations.

Resistance marker presence/absence is often discordant with antimicrobial resistance phenotype

**Figure:**
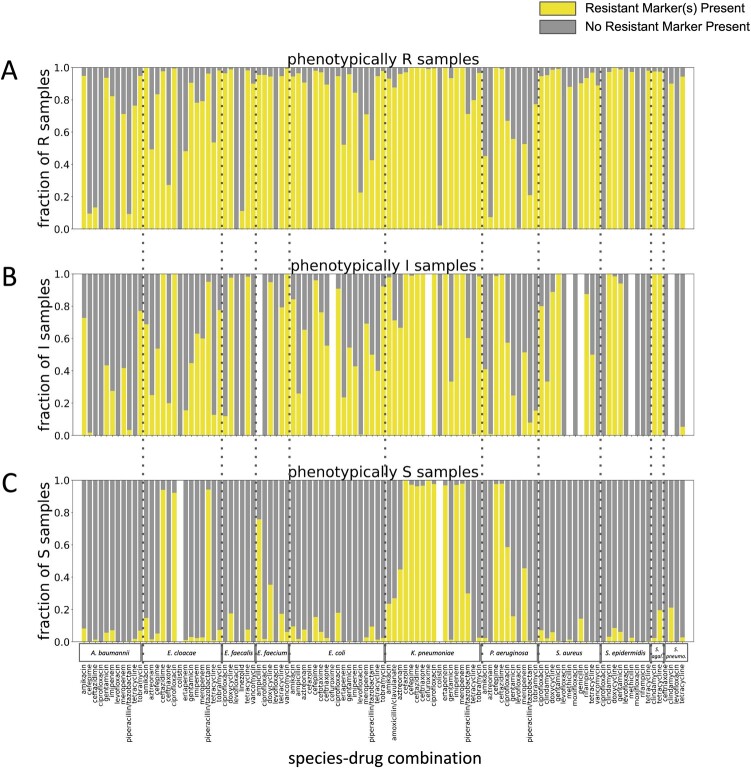

Panels A, B, and C show the proportion of samples with or without relevant resistance markers in resistant (R), intermediate (I), and susceptible (S) samples respectively. Resistance markers relevant to each drug were detected using ResFinder. We evaluated species/drug combinations relevant to bloodstream infections. Overall, ResFinder detected a resistance marker in 72.3% of resistant, 50.3% of intermediate, and 14.2% of susceptible sample-drug combinations.

Comparison of balanced accuracy between Keynome gAST and ResFinder

**Figure:**
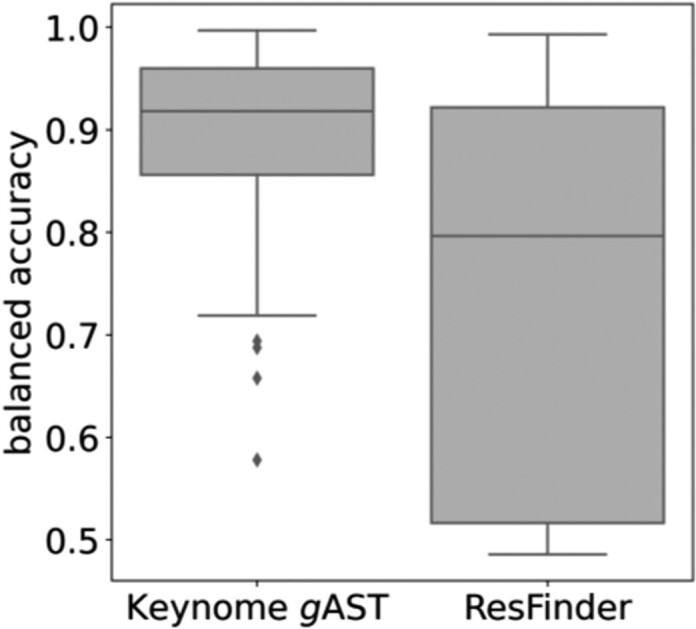

Boxplots compare balanced accuracy across bloodstream relevant species/drug combinations between Keynome gAST and ResFinder. Each box covers the 1st through 3rd quartile, and the horizontal line within the box represents the median. ResFinder performance varied widely across species/drug combinations, with a median balanced accuracy of 80% (52%-92%, 1st and 3rd quartiles). Keynome gAST had a median balanced accuracy of 92% (87%-96%, 1st and 3rd quartiles), and was superior to ResFinder in 69% and equivalent to ResFinder in 28% of species/drug combinations.

**Conclusion:**

The lack of robust correlation between resistance markers and true phenotype highlights the limitations of predicting resistance/susceptibility based on the use of curated resistance markers. A more flexible machine learning approach that accesses the entire bacterial genome allows for complex combinations of genomic regions to inform AST prediction.

**Disclosures:**

**Arolyn Conwill, PhD**, Day Zero Diagnostics: salary|Day Zero Diagnostics: Ownership Interest **Mohamad Sater, PhD**, Day Zero Diagnostics: Employee|Day Zero Diagnostics: Stocks/Bonds **Nicholas Worley, PhD**, Day Zero Diagnostics: Salary|Day Zero Diagnostics: Ownership Interest **Jason Wittenbach, PhD**, Day Zero Diagnostics: Salary|Day Zero Diagnostics: Ownership Interest **Miriam H. Huntley, PhD**, Day Zero Diagnostics: Salary|Day Zero Diagnostics: Ownership Interest

